# Venous Arterialization-Based Extracorporeal Perfusion for Chronic Limb-Threatening Ischemia: A Retrospective Comparative Cohort Study

**DOI:** 10.3390/jcm14248898

**Published:** 2025-12-16

**Authors:** Lei Gao, Xinyuan Qin, Tianbo Li, Boya Li, Jiangning Wang

**Affiliations:** Orthopedic Department, Capital Medical University Affiliated Beijing Shijitan Hospital, No. 10 Yangfangdian Tieyi Road, Haidian District, Beijing 100038, China; gaolei3337@bjsjth.cn (L.G.); qinxinyuan3456@bjsjth.cn (X.Q.); litianbo3285@bjsjth.cn (T.L.); liboya246@126.com (B.L.)

**Keywords:** chronic limb-threatening ischemia, venous arterialization, extracorporeal perfusion, ankle-brachial index, transcutaneous oxygen pressure, wound healing, limb salvage, Visual Analogue Scale

## Abstract

**Background/Objectives:** Chronic limb-threatening ischemia (CLTI) represents the most severe stage of peripheral arterial disease and is associated with high risks of limb loss. Novel approaches are needed for patients who are not candidates for conventional revascularization. This study is to evaluate the clinical efficacy of a venous arterialization-based extracorporeal perfusion technique in patients with CLTI. **Methods:** A retrospective single-centre, non-randomised comparative cohort study was conducted involving 76 patients with chronic limb-threatening ischemia (CLTI), retrospectively assigned into a perfusion group (*n* = 38) and a control group (*n* = 38), with longitudinal pre-/post-treatment assessments at baseline and Day 7 and 6-month limb-salvage follow-up. Patients in the perfusion group received daily extracorporeal perfusion for 6 h over 7 consecutive days. Clinical efficacy was assessed by comparing pre- and post-treatment changes in ankle–brachial index (ABI), transcutaneous oxygen pressure (TcPO_2_), skin temperature, wound area, and Visual Analogue Scale (VAS) pain scores. Limb salvage rates were recorded at 6-month follow-up. **Results:** The perfusion group exhibited significant improvements in ankle–brachial index (ABI) (increase of 0.20 ± 0.02 vs. 0.02 ± 0.01 in the control group, *p* < 0.001), transcutaneous oxygen pressure (TcPO_2_) (increase of 5.24 ± 0.35 mmHg vs. 0.10 ± 0.04 mmHg, *p* < 0.001), skin temperature (increase of 1.19 ± 0.09 °C vs. 0.02 ± 0.01 °C, *p* < 0.001), The mean wound healing rate at 7 days was significantly higher in the perfusion group (23.16 ± 2.30%) compared to the control group (5.62 ± 1.23%) (*p* < 0.001), and Visual Analogue Scale (VAS) score improvement (3.05 ± 1.01 vs. 1.29 ± 0.61, *p* < 0.001) compared with the control group. The 6-month limb salvage rate was significantly higher in the perfusion group (86.8% vs. 26.3%, *p* < 0.001), complete wound healing was achieved in 57.9% of the perfusion group versus 10.5% of the control group (*p* < 0.001). **Conclusions:** Venous arterialization-based extracorporeal perfusion significantly improves microcirculation and clinical symptoms in CLTI patients and may serve as an effective adjunctive therapy to enhance limb salvage outcomes.

## 1. Introduction

Chronic limb-threatening ischemia (CLTI) is a life- and limb-threatening condition that arises from progressive peripheral arterial occlusive disease, manifesting as ischemic rest pain, non-healing ulcers, and tissue loss. It represents the end stage of peripheral artery disease and is associated with extremely poor outcomes—one-year mortality reaches approximately 25% and major amputation rates approach 30% without successful revascularization [1]. Traditional treatments, including bypass surgery and endovascular interventions, aim to restore blood flow to the ischemic limb. However, a substantial subset of patients are deemed “no-option” due to lack of distal target vessels, diffuse disease, or failed prior interventions. In such cases—often referred to as a “desert foot”—conventional revascularization is not feasible and primary amputation is frequently the only solution [2].

Venous arterialization is a revascularization approach that redirects oxygenated arterial blood into the venous system to improve perfusion in ischemic tissue. This strategy creates an artery-to-vein connection so that oxygen-rich arterial blood can perfuse the distal venous and capillary networks of the ischemic limb [3]. By delivering oxygenated blood through the venous circulation, venous arterialization can supply the microcirculation and facilitate wound healing even when the distal arteries are entirely occluded [2]. These techniques—including deep vein arterialization (DVA) via surgical arteriovenous bypass or percutaneous arteriovenous fistulas, as well as external high-pressure extracorporeal perfusion—are designed to bypass occluded distal arteries and utilize the venous and capillary networks to deliver oxygenated blood directly to ischemic tissues. For example, in an open surgical DVA, the great saphenous vein can be anastomosed to a proximal artery (such as the popliteal artery) to drive arterialized blood into the foot’s venous arch–a method that has achieved limb salvage rates around 70% in prior reports [2]. Consistent with this, a recent five-patient series of open DVA reported 80% one-year graft patency and limb salvage [4]. Modern percutaneous DVA techniques (e.g., the LimFlow system) enable catheter-based creation of an arteriovenous fistula in the tibial vessels, allowing arterial flow into the tibial veins and plantar venous plexus of the foot [1]. Early clinical studies of percutaneous DVA have shown high technical success (95%) and encouraging limb salvage outcomes in otherwise inoperable patients [1,3]. By enhancing microcirculatory oxygenation, these approaches aim to promote wound healing, alleviate ischemic pain, and ultimately preserve limb viability in patients with end-stage CLTI.

Among these, the venous arterialization-based extracorporeal perfusion technique leverages a centrifugal pump system—similar to that employed in extracorporeal membrane oxygenation (ECMO)—to withdraw deoxygenated venous blood from the patient’s circulation. The blood is oxygenated via an external membrane lung (oxygenator), transforming it into oxygen-rich arterialized blood. This oxygenated blood is then reinfused into the venous system of the ischemic limb, enabling retrograde perfusion of the capillary bed through the venous network. As a result, the venous system of the affected limb carries blood with arterial-level oxygen tension, markedly improving local tissue oxygenation. Case reports have demonstrated that isolated limb perfusion with an ECMO-like circuit can temporarily sustain limb viability and perfusion in scenarios where no direct arterial revascularization is possible [5]. This approach is intended to improve both wound healing outcomes and limb salvage in patients with critical limb ischemia who are not candidates for conventional revascularization.

Currently, venous arterialization is regarded as a last-resort limb salvage technique for CLTI patients ineligible for traditional revascularization [6,7]. In this context, we retrospectively evaluated the clinical efficacy of a venous arterialization-based extracorporeal perfusion system in improving tissue perfusion, wound healing, pain relief, and 6-month limb-salvage rates in patients with no surgical or interventional options.

## 2. Methods

### 2.1. Study Design and Population

This was a retrospective observational comparative cohort including 76 consecutive patients with CLTI. Patients received either venous arterialization-based extracorporeal perfusion or standard care, with within-patient repeated measures (baseline and Day 7) and 6-month limb-salvage follow-up.

Treatment allocation was non-random and reflected routine clinical decision-making. Patients with no-option CLTI were evaluated by the multidisciplinary team; venous arterialization-based extracorporeal perfusion was offered when venous access was technically feasible, hemodynamic status was acceptable, and informed consent was provided. Patients who declined perfusion, had contraindications to extracorporeal circulation/anticoagulation, or lacked feasible access received standardized conservative therapy and comprised the control cohort.

This study was approved by the Ethics Committee of Capital Medical University Affiliated Beijing Shijitan Hospital (Approval No.sjtkyll-lx-2017(6)). Written informed consent was obtained from all participants, and all personal data were anonymized to ensure confidentiality. Clinical data were retrospectively collected from the Hospital Information System (HIS) of Capital Medical University Affiliated Beijing Shijitan Hospital.

Prior to data extraction, a standardized data collection protocol was developed. Variable definitions, data points to be collected, and coding methods were established and provided to the data extractor to ensure consistency and minimize bias during the retrospective chart review.

Inclusion criteria were as follows:

(1) Persistent rest pain in the affected limb lasting for more than 2 weeks;
(2) Ankle–brachial index (ABI) ≤ 0.40 and transcutaneous oxygen pressure (TcPO_2_) at the distal foot ≤ 30 mmHg;
(3) Absence or severely diminished blood flow signal in pedal arteries (e.g., dorsalis pedis artery, posterior tibial artery) on Doppler ultrasound, defined as a peak systolic velocity < 10 cm/s;
(4) Presence of an ischemic wound with an area between 5–10 cm^2^.

Exclusion criteria included:

(1) Acute phase of myocardial infarction or heart failure, severe hepatic or renal dysfunction, or end-stage renal disease requiring maintenance hemodialysis;
(2) Peripheral vascular diseases such as Takayasu arteritis or other systemic vasculitides;
(3) Severe infection, diabetic ketoacidosis, or other acute metabolic decompensations.

### 2.2. Procedures

#### 2.2.1. Standard Conservative Treatment

All patients in both groups received routine conservative therapy as part of their standard care protocol. Upon admission, wound exudates were collected for bacterial culture and sensitivity testing, and targeted antibiotics were administered accordingly to control local and systemic infections. In addition to antimicrobial treatment, patients were prescribed vasoactive agents to enhance peripheral circulation, analgesics to relieve ischemic pain. Nutritional support was provided to promote tissue repair and overall metabolic balance, with emphasis on adequate protein and caloric intake. Local wound care was individualized based on wound severity and included mechanical or surgical debridement of necrotic tissue, followed by regular dressing changes using sterile techniques. Advanced dressings (e.g., hydrocolloid or silver-containing materials) were employed when necessary to optimize the wound environment and support granulation tissue formation.

#### 2.2.2. Venous Arterialization-Based Extracorporeal Perfusion Protocol

Patients in the perfusion group received daily extracorporeal perfusion for 6 h over 7 consecutive days.

Membrane oxygenation system: Extracorporeal perfusion was delivered using a roller-pump circuit coupled to a hollow-fiber (Polythene) membrane oxygenator [Console: JUN 55X/JUNKEN MEDICAL, Tokyo, Japan; Oxygenator: Hollow Fiber Oxygenator (infantile type)/XIJIAN MEDICAL, Xi’an, China].

**Figure 1:**
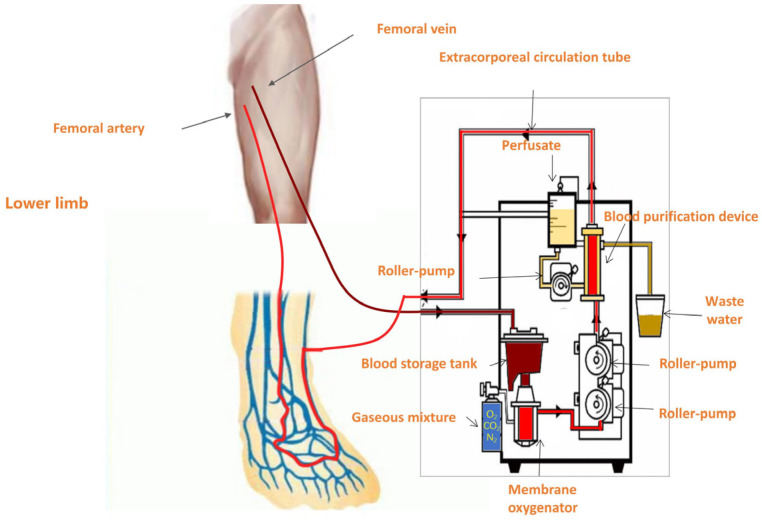
Schematic Diagram of Venous Arterialization-Based Extracorporeal Perfusion.

Under local anesthesia with 1% lidocaine at the puncture site, a 12F venous sheath was inserted into the contralateral femoral vein using the Seldinger technique. This sheath served as the inflow port of the extracorporeal membrane oxygenation-like perfusion circuit. Venous blood was drained from the patient and oxygenated extracorporeally via a membrane lung (oxygenator), thereby generating oxygen-rich arterialized blood to be recirculated. For the target limb, a 6F venous sheath was introduced into the distal segment of a superficial vein (typically the great saphenous vein near the dorsum of the foot) in an antegrade direction. This sheath functioned as the outflow port, delivering oxygenated blood directly into the distal venous system of the ischemic limb. Vascular access was venous-only: a 12F sheath in the contralateral femoral vein for drainage and a 6F sheath in a distal superficial vein of the target limb (typically the great saphenous vein near the dorsum) for reinfusion. No arterial cannulation (e.g., tibial or pedal arteries) was performed. At the initiation of perfusion, a pneumatic tourniquet was applied to the mid-thigh (proximal femur) region of the affected limb. To facilitate retrograde perfusion while preserving native arterial inflow, the tourniquet pressure was maintained between 30–60 mmHg, a range below the arterial occlusion threshold yet above superficial venous pressure. This setup enabled effective occlusion of proximal superficial venous outflow, redirecting the oxygenated perfusate into deep veins via communicating (perforator) branches, thereby enhancing microvascular oxygen delivery to ischemic tissues. The perfusion circuit thus established a retrograde flow of arterialized blood into the venous network of the foot, promoting tissue oxygenation through the extensive superficial–deep venous communication system (Figure 1).

Technical success was defined as successful cannulation of the planned venous inflow/outflow sites and initiation of the extracorporeal perfusion protocol on Day 1 with stable circuit flows. The analytic perfusion cohort comprised only cases meeting this definition.

#### 2.2.3. Perfusion Circuit Parameters and Safety Protocol

The extracorporeal circuit consisted of a roller pump coupled to a hollow-fiber membrane oxygenator, with venous drainage via a 12F contralateral femoral sheath and distal reinfusion via a 6F superficial foot vein; a pneumatic thigh tourniquet (30–60 mmHg) was used to favor retrograde deep-venous perfusion.

Typical blood-flow targets were 60–80 mL/min at initiation, titrated to 100–120 mL/min as tolerated; we avoided flows > 150 mL/min through the 6F reinfusion sheath. Reinfusion-line pressure was maintained <80–90 mmHg; flow was reduced if this threshold was exceeded.

Gas and temperature settings included FiO_2_ = 1.0 with sweep gas 1–3 L/min (initially 2 L/min), adjusted to keep post-oxygenator PaO_2_ > 200 mmHg on intermittent blood-gas checks; the heat-exchanger was set to 36–37 °C. Anticoagulation used intravenous unfractionated heparin (50–70 U/kg bolus before cannulation), followed by 10–15 U/kg/h, titrated to ACT 180–220 s during the first hour and maintenance aPTT 50–70 s (or anti-Xa 0.3–0.5 IU/mL) thereafter. Safety laboratory thresholds included platelets > 80 × 10^9^/L and fibrinogen > 1.5 g/L. ACT was measured hourly for the first 3 h, then every 2–4 h.

Haemodynamic tolerance and stop rules: non-invasive BP and HR were continuously monitored; flow was reduced or temporarily paused if mean arterial pressure (MAP) decreased ≥10% from baseline, if distal venous engorgement/ooze occurred, or if ischemic pain acutely worsened. The tourniquet was adjusted within 30–60 mmHg to optimize deep-venous redistribution.

Oxygenator surveillance: transmembrane pressure (TMP) was recorded hourly; TMP > 250 mmHg or a rising trend prompted elective oxygenator exchange and anticoagulation reassessment.

### 2.3. Assessment Indicators

Clinical efficacy was evaluated using a comprehensive set of hemodynamic, physiological, and wound-healing parameters, as well as patient-reported pain scores and hard clinical endpoints. Assessments were performed at baseline (pre-treatment) and on Day 7 (post-treatment), unless otherwise specified. All post-treatment assessments were obtained within 12 h after completion of the seventh perfusion session, following discontinuation of extracorporeal circulation, to represent the cumulative treatment effect rather than values during active perfusion.

Ankle–Brachial Index (ABI): ABI was calculated as the ratio of the higher ankle systolic pressure (posterior tibial or dorsalis pedis artery) to the higher brachial artery systolic pressure. A change ≥ 0.10 was considered clinically meaningful.

Transcutaneous Oxygen Pressure (TcPO_2_): TcPO_2_ is a noninvasive measure of the partial pressure of oxygen diffusing through the skin from the capillaries. It reflects local tissue oxygenation and microvascular perfusion. In CLTI, a baseline TcPO_2_ < 20 mmHg correlates with high risk of nonhealing ulcers and amputation. A ΔTcPO_2_ ≥ 5 mmHg was predefined as a significant improvement.

Skin Temperature: Recorded using an infrared dermal thermometer at three standardized sites (dorsum, medial midfoot, and plantar surface) and averaged. An increase ≥ 1 °C was deemed clinically significant.

Wound Healing Rate: (wound area_pre_ − wound area_post_)/wound area_pre_. The wound healing rate represented the percentage reduction in wound area compared with baseline, reflecting early granulation and perfusion improvement rather than complete epithelial closure.

Visual Analogue Scale (VAS) Pain Score: Patients rated their rest pain on a 0–10 scale (0 = no pain; 10 = worst imaginable pain). A decrease of ≥2 points was considered a clinically important pain relief.

**Figure 2:**
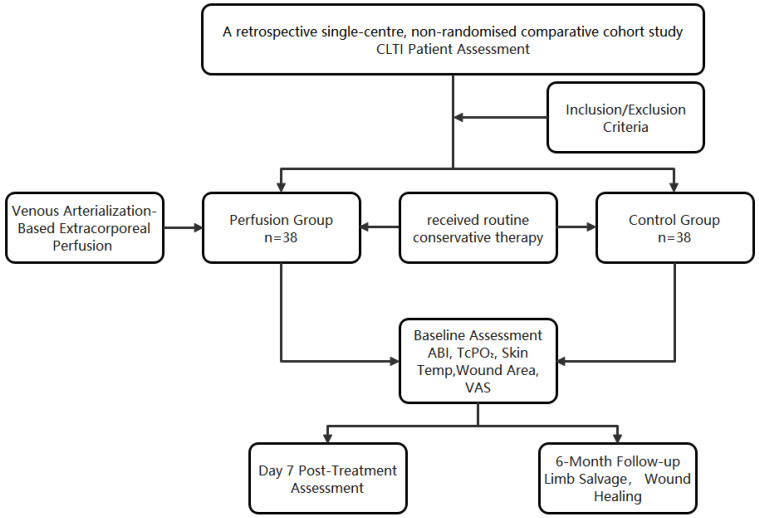
Flowchart of patient inclusion, allocation, and analysis.

Limb-Salvage Rate: Defined as the proportion of patients alive without above-ankle amputation at 6 months post-treatment. Events (major amputations or mortality) were confirmed through clinical records and telephone follow-up (Figure 2).

### 2.4. Statistical Analyses

Continuous variables were expressed as mean ± SD and compared using paired or independent *t*-tests; categorical outcomes (e.g., limb salvage) were compared using chi-square tests. A two-tailed *p* < 0.05 was considered statistically significant.

## 3. Results

A total of 76 patients diagnosed with chronic limb-threatening ischemia (CLTI) and treated between January 2019 and January 2020 were included in this retrospective comparative cohort study. Patients were divided into two groups (perfusion group: *n* = 38 and control group: *n* = 38) based on the therapeutic modality received (Table 1). There were no significant differences between the two groups in baseline characteristics, including age, sex, diabetes history, or other clinical variables (*p* > 0.05), indicating comparability.

Following the 7-day treatment period, the perfusion group showed significant improvements across all clinical indicators compared with the control group, including smaller wound area, higher ABI and TcPO_2_, elevated skin temperature, and reduced pain scores. These changes indicate enhanced tissue perfusion, oxygenation, and early wound healing (Table 2).

**Figure 3:**
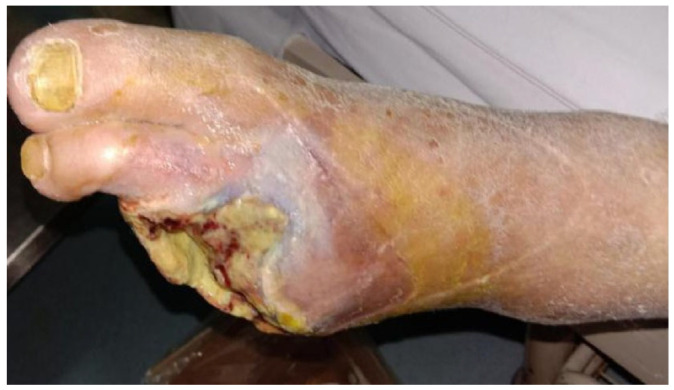
Representative wound appearance in the perfusion group at baseline (Day 0), showing ischemic ulcer with necrotic margins and poor granulation.

**Figure 4:**
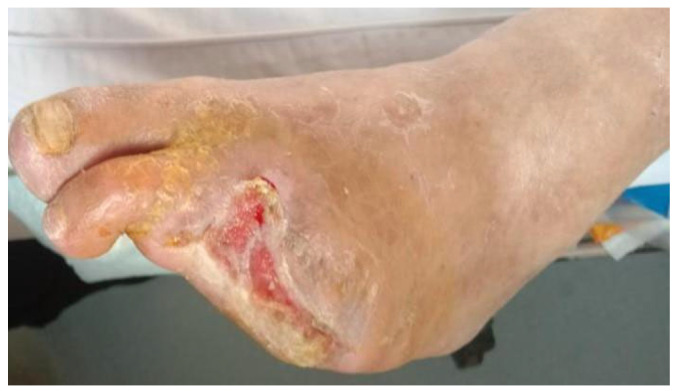
The same wound after 7 days of venous arterialization-based extracorporeal perfusion, demonstrating decreased necrotic tissue, new granulation growth, and reduced wound area.

After 7 days of treatment, patients in the perfusion group exhibited significantly greater clinical improvements compared with the control group. Increases in ABI, TcPO_2_, and skin temperature, together with reduced wound area and pain scores, confirmed the restoration of microcirculatory perfusion and accelerated wound recovery (Table 3) (Figure 3 and Figure 4).

Complications were infrequent and all were minor, no unplanned surgical/interventional procedures, no ICU care). We observed one case of access-site bleeding/hematoma at the contralateral femoral venous sheath; this was managed with immediate manual compression, a pressure dressing for 24–48 h, and temporary down-titration/brief pause of anticoagulation, with complete resolution and no sequelae. Two patients developed oozing at the distal reinfusion puncture site; both were treated with local compression, limb elevation and a light compression wrap, absorbent/alginate dressing changes, and short-term anticoagulation adjustment, resolving within 48–72 h. One patient had a puncture-site infection that responded to local wound care plus a short course of empiric antibiotics per institutional protocol; no drainage procedure was required. No cases of limb venous thrombosis were recorded. Three oxygenator exchanges were performed because of rising transmembrane pressure or suspected thrombus formation; in each instance the circuit was electively exchanged and anticoagulation titrated per protocol, and perfusion resumed uneventfully. No patient discontinued the 7-day regimen due to these events.

No patient was lost to follow-up at 6 months via hospital records or structured telephone follow-up. Two patients in the perfusion group and three in the control group died from causes unrelated to the perfusion procedure (cardiovascular and renal failure). None of these deaths occurred during the 7-day treatment window. These deaths were counted as “events” (failure of limb salvage) according to the predefined endpoint “alive without above-ankle amputation,” consistent with standard limb-salvage reporting in CLTI studies.

At 6 months post-treatment, limb salvage was achieved in 86.8% (33/38) of patients in the perfusion group, compared to only 26.3% (10/38) in the control group. The difference was statistically significant (χ^2^ = 25.922, *p* < 0.001), indicating that venous arterialization-based extracorporeal perfusion markedly enhances limb preservation in patients with chronic limb-threatening ischemia (CLTI). In addition to limb salvage, a significant improvement in wound healing was observed. At 6 months, 22 out of 38 patients (57.9%) in the perfusion group achieved complete wound closure, whereas only 4 out of 38 (10.5%) in the control group exhibited similar healing. This difference was also statistically significant (χ^2^ = 16.895, *p* < 0.001). These findings suggest that the perfusion technique not only prolongs limb viability but also facilitates meaningful tissue recovery, potentially reducing the need for amputation and improving long-term functional outcomes.

## 4. Discussion

Venous arterialization techniques have shown promise in salvaging limbs in patients with chronic limb-threatening ischemia (CLTI) who lack conventional revascularization options. Recent trials of percutaneous deep vein arterialization (pDVA) report one-year limb salvage rates around 66–70% [8,9]. Open surgical deep vein arterialization (DVA), an older approach involving bypass of a distal artery into the venous circulation, has also demonstrated limb salvage potential. Small series of open DVA have reported 1-year limb salvage rates on the order of 60–80%, though results vary with patient selection [10]. Open DVA yielded primary patency rates of 44–88% at 1 year, substantially higher than the 29–40% patency at 6 months seen with early percutaneous methods [11]. Importantly, even when the created artery-to-vein conduit occludes, limbs can remain viable due to established collateral circulation. A recent office-based pDVA series illustrates this phenomenon: only ~6% of the arteriovenous grafts were patent at 6 months, yet 78.6% of patients avoided major amputation during follow-up [12]. This suggests that venous arterialization promotes angiogenesis and microcirculatory perfusion sufficient for wound healing and limb survival, even if the initial shunt closes.

Wound healing with venous arterialization is typically protracted. Unlike surgical bypass, which can restore direct arterial flow and expedite ulcer healing, pDVA often requires 8–12 weeks for neovascularization before significant clinical improvement is evident [13]. Early after the procedure, wounds may appear stagnant or only partially improved; however, by 6–12 months many patients achieve stable wound closure [8]. In PROMISE II, 92% of patients demonstrated progressive ulcer healing and 45% had fully healed wounds at one year. Similarly, a meta-analysis found an overall complete wound healing rate of ~46% at 12 months post-pDVA. These data underscore that venous arterialization can ultimately facilitate meaningful wound healing in roughly half of no-option patients, though continual wound care and patience are required [8,13]. In this context, our observed 6-month complete wound healing rate of 57.9% compares favorably and may reflect a relatively accelerated healing trajectory, possibly due to the high-concentration oxygen delivery and controlled flow environment provided by the extracorporeal circuit. This system mimics ECMO-like conditions, where temporary, high-oxygen perfusion through the venous network may enhance capillary recruitment and tissue oxygenation more efficiently than standard pDVA. Thus, our data align with the broader literature while also suggesting that extracorporeal perfusion could represent a viable, possibly more rapid alternative to pDVA in select no-option CLTI patients.

Objective perfusion indices provide additional evidence of the efficacy of these novel techniques. Conventional revascularization via bypass or angioplasty typically produces a marked rise in ankle–brachial index (ABI) by restoring large-artery inflow. In contrast, venous arterialization routes blood through the venous plexus, so ABI improvements are often blunted. Many pDVA studies report minimal changes in ABI post-procedure (since pedal arteries remain occluded), focusing instead on microcirculatory measures like toe pressure or transcutaneous oxygen tension (TcPO_2_). Notably, one extracorporeal perfusion approach in Australia achieved a dramatic ABI increase from 0.04 at baseline to 0.63 after treatment (*p* < 0.05). Our results also demonstrated significant improvements in key physiological parameters. The perfusion group showed a mean increase in ankle–brachial index (ABI) of 0.19 ± 0.02 compared to only 0.02 ± 0.01 in controls, which is consistent with previous extracorporeal perfusion studies. In particular, a study by Khin et al. using hypertensive extracorporeal limb perfusion observed a post-treatment ABI increase from 0.04 to 0.63 [14]. This marked improvement in ABI, combined with significant enhancements in skin temperature and TcPO_2_ in our cohort, indicates that venous arterialization-based perfusion can restore both macro- and micro-circulatory function, even when traditional arterial pathways are not amenable to intervention.

From a hemodynamic perspective, the most suitable venous conduits for such perfusion are those lacking valves, as the absence of valvular resistance permits unobstructed retrograde flow of oxygenated blood toward the distal venous plexus. This valveless architecture facilitates a more uniform distribution of the perfusate through the communicating and deep venous networks, optimizing microcirculatory oxygen delivery. Furthermore, the perfusion process itself can provoke venous diameter dilatation secondary to increased intraluminal pressure and shear stress, which enhances downstream arterial-like flow and promotes endothelial adaptation. These synergistic mechanisms—valveless conduit selection and perfusion-induced venous remodeling—collectively favor improved tissue oxygenation and distal perfusion, explaining the observed rise in ABI, TcPO_2_, and skin temperature in our study.

Early evidence suggests that venous arterialization techniques are generally safe when performed in experienced centers, but they are not without risks. Procedure-related complications for percutaneous DVA have been reported in roughly 10–15% of patients [15]. These include issues like access-site bleeding, hematoma, or distal embolization, as well as venous dissection or thrombosis in the newly created fistula [14,15]. Postprocedural venous hypertension in the foot is a unique concern: patients commonly experience swelling and venous engorgement, which can initially worsen pain or cause wound ooze in the weeks after arterialization [13]. Careful management (elevating the limb, compression therapy, etc.) is needed to mitigate venous congestion while collateral pathways develop. In most cases, this early pain and edema subside over 2–3 months as the venous system adapts. In keeping with our venous-only access strategy, the safety profile was dominated by venous access-related issues (groin hematoma, distal puncture-site oozing) and anticoagulation-related constraints rather than small arterial vessel injury. Most events were minor and manageable with hemostasis, limb elevation/compression, local wound care, and protocolized anticoagulation monitoring; isolated major events required targeted intervention. These observations align with prior reports of venous arterialization techniques, in which access bleeding/hematoma and venous thrombosis predominate, and highlight the importance of meticulous ultrasound-guided cannulation, standardized hemostasis, and early management of venous congestion.”

In our real-world experience, venous arterialization-based extracorporeal perfusion used venous-only access (12 F contralateral femoral venous drainage and 6 F distal superficial venous reinfusion), so small tibial/pedal arteries were not instrumented; observed issues therefore centered on venous access and anticoagulation rather than arterial injury. Consistent with the literature’s emphasis on careful management of venous congestion, our protocol relied on ultrasound-guided cannulation, meticulous hemostasis, elevation/compression, and standardized anticoagulation monitoring to mitigate bleeding and distal oozing while collateral pathways developed.

Another challenge is the durability of the arteriovenous conduit. As noted, primary patency of pDVA channels is low–often only ~20–30% at 6 months. Consequently, reinterventions are frequent. Approximately one-third to nearly half of patients undergo repeat endovascular interventions (balloon dilatation, stenting, or thrombectomy of the fistula) within the first year to maintain perfusion [8,15]. Despite this, the fact that limb salvage can far exceed conduit patency underscores that even transient patency may confer lasting biologic benefit (via angiogenesis). Regarding systemic effects, venous arterialization is well tolerated hemodynamically since it leverages low-pressure outflow; the all-cause mortality in these high-risk CLTI cohorts is on the order of 15–20% at 1 year, driven largely by comorbidities rather than the procedure itself. This is comparable to outcomes in advanced CLI patients after conventional therapies.

While venous arterialization has expanded the armamentarium for no-option CLTI, it is generally considered adjunctive or salvage therapy rather than a replacement for conventional revascularization. In patients who have re-constructible distal arteries, standard surgical bypass or percutaneous transluminal angioplasty (PTA) remain first-line therapies, offering superior long-term patency and well-established efficacy. For example, the recent BEST-CLI trial demonstrated that in CLTI patients with usable saphenous vein grafts, open bypass achieved significantly better limb outcomes than endovascular treatment–over a median ~3-year follow-up, the surgical arm had fewer major amputations (≈9% vs. 15%) and fewer major reinterventions. One-year limb-salvage rates with successful bypass are often reported in the 80–90% range [16].

Comparisons between domestic and international studies reveal certain differences in the application of venous arterialization. Internationally, percutaneous techniques such as the LimFlow system have gained increasing acceptance, with multicenter trials demonstrating both safety and efficacy [17,18]. In contrast, domestic studies have explored extracorporeal perfusion models using ECMO-like circuits, reflecting a unique approach to oxygenating the venous system in ischemic limbs. Both strategies share the common goal of improving perfusion in no-option patients, and both have demonstrated substantial limb salvage benefits.

This study has several important limitations. First, it was a retrospective, single-center analysis, which introduces risks of selection bias, incomplete data capture, and unmeasured or residual confounding. Treatment allocation was not randomized, and concomitant therapies could not be standardized, limiting causal inference. Second, formal between-group angiographic comparison was not feasible in our cohort because diagnostic angiography was not obtained systematically in both groups during the real-world implementation phase, and imaging—when performed—occurred at non-uniform, clinically driven time points. Third, although we expanded Table 1 to include comorbidities (diabetes duration, cardiac function, renal insufficiency), the study was not powered to assess their independent effects on wound healing. Fourth, perfusion indices were recorded only at baseline and day 7, which may not capture intermediate or longer-term dynamics, and follow-up was limited to 6 months, potentially underestimating late outcomes or complications. Because this was a retrospective, real-world study, most patients were discharged soon after the 7-day treatment course and did not return for standardized 30-day follow-up hemodynamic testing. The follow-up data available at 30 days mainly concerned clinical outcomes such as wound condition and amputation status recorded through hospital readmission or outpatient review, but systematic physiologic reassessment (ABI, TcPO_2_, skin temperature) was not routinely performed in all cases. Therefore, only the Day-7 data—obtained under uniform inpatient conditions—could be analyzed reliably and comparably. Fifth, the sample size was relatively small and reflected real-world caseloads during the early implementation phase, which restricts generalizability. Sixth, this study was conducted as a retrospective, single-center analysis during the early clinical implementation phase of venous arterialization-based extracorporeal perfusion. As such, non-randomized inclusion and observational design were inherent constraints. Because this was a retrospective non-randomized analysis conducted during the early clinical adoption phase, potential selection bias and unmeasured confounding cannot be fully excluded. Future prospective, multicenter randomized studies are planned to further validate these preliminary findings. Finally, because this was a feasibility and hypothesis-generating analysis in a salvage population, further prospective, multicenter trials with standardized imaging, laboratory protocols, and longer-term follow-up are required to validate these findings and refine patient selection criteria”.

## 5. Conclusions

In conclusion, our study supports venous arterialization-based extracorporeal perfusion as a feasible and effective salvage therapy for CLTI patients who lack traditional revascularization options. This technique significantly improves perfusion indices, reduces ischemic pain, and enhances limb salvage rates. Although long-term conduit patency remains a challenge and close postoperative monitoring is essential, venous arterialization provides a valuable opportunity to preserve limb function in patients who would otherwise face inevitable amputation. Future studies with larger sample sizes and extended follow-up are warranted to further validate these findings and refine patient selection criteria.

## Figures and Tables

**Table 1:** General patient information (*n*, Mean ± SD).

Variable	Perfusion Group *n* = 38	Control Group *n* = 38	*p*-Value
**Age (years)**	70.03 ± 6.99	72.45 ± 6.61	0.125
**Sex**			
Male	20/38 (52.6%)	23/38 (60.5%)	0.643
Female	18/38 (47.4%)	15/38 (39.5%)	
**Medical history**			
Diabetes: NO	8/38 (21.1%)	5/38 (13.2%)	0.542
YES	30/38 (78.9%)	33/38 (86.8%)	
Coronary heart disease: NO	23/38 (60.5%)	20/38 (52.6%)	0.643
YES	15/38 (39.5%)	18/38 (47.4%)	
Cardiac function NYHA class: I–II	33/38 (86.8%)	31/38 (81.6%)	0.753
III–IV	5/38 (13.2%)	7/38 (18.4%)	
Renal insufficiency: NO	12/38 (31.6%)	16/38 (42.1%)	0.476
YES	26/38 (68.4%)	22/38 (57.9%)	
**Previous procedures/interventions**			
Prior endovascular revascularization: NO	24/38 (63.2%)	19/38 (50.0%)	0.355
YES	14/38 (36.8%)	19/38 (50.0%)	
Prior surgical bypass: NO	33/38 (86.8%)	28/38 (73.7%)	0.249
YES	5/38 (13.2%)	10/38 (26.3%)	
Prior debridement: NO	25/38 (65.8%)	23/38 (60.5%)	0.812
YES	13/38 (34.2%)	15/38 (39.5%)	
**Wound Duration (days)**	50.26 ± 4.68	49.92 ± 4.62	0.7492
**Wound Area (cm^2^)**	8.61 ± 0.50	8.79 ± 0.46	0.0988
**ABI**	0.33 ± 0.02	0.34 ± 0.02	0.0518
**Skin-Temp. (°C)**	28.08 ± 0.45	28.19 ± 0.36	0.2525
**TcPO_2_ (mmHg)**	24.78 ± 0.90	24.96 ± 1.03	0.4341
**VAS**	7.00 ± 1.09	7.08 ± 1.19	0.7643
**Albumin (g/L)**	34.74 ± 4.01	35.14 ± 3.68	0.6554
**Total Chol (mmol/L)**	4.84 ± 0.53	4.65 ± 0.65	0.147
**LDL-C (mmol/L)**	2.59 ± 0.41	2.58 ± 0.38	0.8651

Note: Baseline laboratory variables included Albumin (g/L), LDL-cholesterol (LDL-C, mmol/L), and Total Chol (TC, mmol/L) measured by the hospital central laboratory using standard enzymatic methods. The first available sample within 24 h of admission (pre-treatment) was used as baseline.

**Table 2:** Comparison of Post-Treatment Clinical Parameters Between Perfusion and Control Groups (Mean ± SD, *t*-test).

Variable	Perfusion Group *n* = 38	Control Group *n* = 38	t-Statistic	*p*-Value
ABI	0.53 ± 0.03	0.36 ± 0.02	27.35	<0.001
Skin-Temp. (°C)	29.31 ± 0.47	28.21 ± 0.36	11.361	<0.001
TcPO_2_ (mmHg)	30.04 ± 0.92	25.06 ± 1.03	22.2	<0.001
Wound Healing Rate (%)	23.16 ± 2.30	5.62 ± 1.23	41.43	<0.001
VAS	3.95 ± 1.01	5.79 ± 1.02	−7.912	<0.001

**Table 3:** Comparison of Post-Treatment Clinical Improvements Between Perfusion and Control Groups (Mean ± SD, *t*-test, *p*-value).

Variable	Perfusion Group *n* = 38	Control Group *n* = 38	Mean Difference	95% CI	t-Statistic	*p*-Value
ABI Change	0.19 ± 0.02	0.02 ± 0.01	0.17	0.17 to 0.18	43.231	<0.001
TcPO_2_ Change	5.26 ± 0.28	0.10 ± 0.05	5.16	5.06 to 5.25	110.08	<0.001
Skin-Temp. Change	1.23 ± 0.11	0.02 ± 0.01	1.21	1.17 to 1.24	68.651	<0.001
VAS Change	3.05 ± 1.01	1.29 ± 0.61	1.76	1.38 to 2.15	9.194	<0.001
Wound Area Change	1.99 ± 0.19	0.49 ± 0.10	1.5	1.43 to 1.57	43.161	<0.001

## Data Availability

The original contributions presented in this study are included in the article. Further inquiries can be directed to the corresponding author(s).
